# “It's that feeling that you can't get away”: Motherhood, gender inequality and the stress process during extreme events

**DOI:** 10.1111/bjso.12856

**Published:** 2025-01-29

**Authors:** Evangelos Ntontis, Jennifer Monkhouse, Natalie Stokes‐Guizani, Aida Malovic, Patricio Saavedra

**Affiliations:** ^1^ School of Psychology and Counselling The Open University Milton Keynes UK; ^2^ Centre for Health Services Studies, University of Kent Canterbury UK; ^3^ School of Psychology and Life Sciences Canterbury Christ Church University Canterbury UK; ^4^ Instituto de Ciencias Sociales Universidad de O'Higgins Rancagua Chile

**Keywords:** COVID‐19, extreme events, gender, mother, secondary stressors, stress

## Abstract

The impacts of extreme events can intersect with pre‐disaster systemic inequalities and deficiencies, exacerbating distress. This paper contributes to the existing literature by exploring the psychosocial processes through which stressors become traumatic during an extreme event. It does so by focusing on how mothers of children and/or adolescents in the United Kingdom experienced the COVID‐19 pandemic. First, qualitative interviews (*N* = 15) showed that participants experienced a cluster of stressors stemming from their workplaces, partners, children's behaviours and homeschooling, which caused a sense of overload and captivity, reducing their quality of life. However, individual, interpersonal and collective forms of coping were reported. Second, quantitative survey data (*N* = 621) showed that the relationship between stressors and perceived stress was mediated by feelings of overload due to excessive identity‐related tasks and caregiving responsibilities. Moreover, community identification was associated with reduced overload and perceived stress. Overall, during extreme events, people can experience distress due to being overloaded by and trapped in particular identities and identity‐related tasks, unable to perform other aspects of their social selves. We argue that social psychological analyses can be useful in tracing the complex impacts of extreme events across a range of systems and levels of analysis.

## INTRODUCTION

Social identity processes can shape how people perceive and respond to times of crisis, which can cause major life transitions, identity change or stress, to name a few (Haslam et al., 2021; Haslam & Reicher, 2006; Muldoon, 2024; Muldoon et al., 2019). Our paper further delves into the psychosocial processes through which stressors become traumatic during extreme events. Extreme events such as floods, earthquakes, hurricanes and pandemics can be pivotal, life‐changing moments for those affected. Not only can they cause distress and mental ill health, but such incidents cut deeply through the fabric of social life by altering spaces, displacing communities and causing significant changes to people's routines and sense of self (Erikson, 1976, 1994; Erikson & Peek, 2022; Kaniasty & Norris, 1999). Considering the multilayered impacts of extreme events, our aim requires us to move beyond merely mapping the stressors that people face and to investigate how social identities and the dynamics of social relationships within people's situated realities can either interact with other stressors or comprise additional stressors themselves, exacerbating distress and potentially becoming traumatic.

In this paper, we use semi‐structured interview and survey data to examine how mothers of children and/or adolescents experienced the early stages of the COVID‐19 pandemic. Our analyses serve to highlight the complex psychosocial impacts of long‐lasting extreme events on people's sense of self and well‐being, particularly when the latter are already positioned in precarious positions due to social inequalities.

### Gender inequality, motherhood, caregiving and extreme events

Income, gender and health inequalities can exacerbate negative health outcomes (Davidson, 2014; Nomura et al., 2016; Wilkinson & Pickett, 2010). This also applies to extreme events, where people who are older, female, poor, less educated or belong to ethnic minorities can suffer more by receiving less support or by being excluded from disaster communities, since these social categories are linked to existing and enduring patterns of inequality (Kaniasty & Norris, 1995, 1999; Norris et al., 2002; Ntontis, Drury, Amlot, et al., 2020; Ntontis, Drury, Amlôt, et al., 2020).

Gender inequalities more specifically position women in rather precarious positions in the social order. Household duties are still widely perceived to be women's responsibilities (Arntz et al., 2020), with the distribution of domestic duties not having shifted significantly over the last few decades despite more women taking up professional roles (Lachance‐Grzela & Bouchard, 2010). Parenting systems based on gender inequality can affect mothers' stress (Medina & Magnuson, 2009), with mothers who are married, employed or have young children being most at risk (Benin & Keith, 1995; Lease, 1999). Moreover, apart from a cognitive self‐definition, the social identity of ‘mother’ inscribes particular obligations of caregiving towards one's child and, in general, the younger the child is, the higher the demands for caregiving can be. Particularly for mothers with high levels of caring responsibilities, stress can develop because of social disconnection and isolation (Demirtepe‐Saygılı & Bozo, 2010; Wang et al., 2020). Thus, the multifaceted impacts of gender inequality can create a double victimization, with some people suffering because they are both women and mothers.

Caregiving itself, a practice associated with motherhood (and parenthood in general), can be a traumatic experience due to high material and psychosocial demands. It can make the providers of support feel trapped in their role as caregivers, leading to the emergence of psychosocial and material problems. These include conflicting roles and role strains, a loss of self, financial constraints, limitations in opportunities for socialization or a sense of role overload and role captivity (Pearlin, 1989; Pearlin et al., 1997). Role overload is a primary negative outcome of caregiving and refers to the sense of feeling overwhelmed and overburdened with particular role tasks. Role captivity is a secondary intrapsychic outcome of caregiving and refers to a sense that one is constrained to a particular role without being able to experience or perform other aspects of the self (Pearlin et al., 1990). Considering that extreme events can cause people to lose their social connections and potentially entrap them in particular environments or sets of social relationships often shaped by inequality, the notions of overload and captivity can help us better understand how extreme events become traumatic.

All of the aforementioned patterns can be observed during extreme events, where woman often experience higher distress (Norris et al., 2002), and this was the case with the COVID‐19 pandemic. Many women were distressed because of having to manage their children's education or caring for elderly in parallel with employment, highlighting the health and financial burdens of emotional and caring labour (Arntz et al., 2020; Goldin, 2022). Constraints in mobility also increased exposure to intimate partner violence (Almeida et al., 2020; Burki, 2020), whereas higher but disproportionate caring responsibilities demonstrated the accentuated gender disparities during the pandemic, with employed women or single parents experiencing more negative mental health outcomes (Almeida et al., 2020).

### Stress and social identity processes in extreme events

Most of the people affected by extreme events will not exhibit psychopathology (Goldmann & Galea, 2014; Stancombe, Stokes, et al., 2024) but rather symptoms of distress. Distress refers to negative, overwhelming “*experiences and feelings of people after external events that challenge their tolerance and adaptation*” (Department of Health, 2009, p. 20) and often decreases over time through social support (Stancombe, Williams, & Kemp, 2024; Williams et al., 2014).

Stressors are events, circumstances, responses and attitudes that can trigger distress, or are factors that can cause tension due to being perceived as excessive by those affected (Stokols, 1985), challenging people's perceived capacity to cope with them (Lazarus & Folkman, 1984). Stressors in extreme events can be understood as belonging in one of two categories, ‘primary’ and ‘secondary’. Primary stressors are “*inherent in particular major incidents, disasters, and emergencies and arising directly from those events*” (Department of Health, 2009, p. 20), reflecting direct exposure to extreme events (e.g. injuries, death, illness, damage). Secondary stressors on the other hand reflect people's pre‐disaster life circumstances (e.g. illness) or social factors (e.g. excessive bureaucracy) that carry over during an extreme event and become additional problems or arise due to inefficient and problematic responses to the extreme event (Ntontis et al., 2024; Ntontis & Zhang, 2024; Williams et al., 2021, 2024). Secondary stressors can cause significant distress. For example, relationship problems, loss of sentimental items, lacking insurance or warnings, or being displaced were associated with increased odds of anxiety, depression and PTSD (Mulchandani et al., 2019; Munro et al., 2017; Tempest et al., 2017). Ntontis et al. (2023) found that women and less affluent people reported more distress and less resilience during the COVID‐19 pandemic because they experienced more secondary stressors like employment concerns, relationship problems, child support or not having access to health care.

Due to their excessive nature and the multiple stressors present, extreme events can affect people in two ways: by causing distress directly or by potentially comprising significant moments of transition and change. Social identity can shape these pathways. First, social identity can affect how people respond to stressful situations by providing a platform for group members to receive social support and by enabling them to resist the influence of stressors (Haslam et al., 2005; Haslam & Reicher, 2006). In extreme events including bombings, flooding, earthquakes and pandemics, shared social identity has been associated with increases in group belonging, expected support, collective efficacy and well‐being (Drury et al., 2016, 2019; Ntontis et al., 2018, 2021, 2023; Ntontis, Drury, Amlot, et al., 2020; Ntontis, Drury, Amlôt, et al., 2020; Ntontis & Zhang, 2024; Stancombe et al., 2022; Vignoles et al., 2021). However, when characterized by negative content and norms that promote risky behaviours, group memberships can become conduits of harm for group members. Similarly, if group members cannot leave the groups, redefine their identities or change the nature of the social relations with other group members, then they can be chronically exposed to stressors and experience stress (Wakefield et al., 2019).

Second, times of transition and crisis can be traumatic as they disrupt people's routines and can cause identity shifts (Haslam et al., 2021). Losing social identities due to the effects of stressors can lead to lower well‐being due to reductions in one's social networks and the availability of social support (Jetten et al., 2009; Praharso et al., 2017). Moreover, not only can trauma be a response to valued identities being undermined, but trauma responses can be shaped by the availability of social resources that can help people to maintain a positive sense of self and to maintain or expand their social connections (Muldoon et al., 2017, 2019, 2023). This is particularly important for disasters and extreme events whereby the impact of the disaster can lead to identity loss not only due to community displacement but also due to loss of valued spaces and items. Nevertheless, the emergence of new identities is commonly observed in disasters (Drury et al., 2019; Ntontis et al., 2019), but equally common are cases of people choosing to distance themselves from identities perceived as traumatic (Erikson, 1994; Ntontis, Drury, Amlot, et al., 2020; Ntontis, Drury, Amlôt, et al., 2020; Ntontis & Zhang, 2024).

### The present paper

Recent theoretical models have highlighted the central role of secondary stressors in mobilizing the stress process (Williams et al., 2021, 2024), with empirical evidence stressing the usefulness of these frameworks in terms of their theoretical and applied potential (Ntontis et al., 2023, 2024; Stancombe et al., 2022, 2023). The aim of this paper is to further develop our understandings of the psychosocial processes through which secondary stressors become distressing during extreme events and to examine the role of community identification. We draw on contemporary models on social identity and stress (e.g. Haslam & Reicher, 2006), identity change (e.g. Haslam et al., 2021; Muldoon, 2024; Muldoon et al., 2019) and the social cure approach (Haslam et al., 2018; Wakefield et al., 2019) as these frameworks examine individual experiences while situating them in wider networks of social relations.

We focus on mothers of children and/or adolescents that experienced the COVID‐19 pandemic, some of which faced double victimization due to gender inequalities: first because of being women, who experience more distress during extreme events, and second because of being mothers, whose material, emotional and caregiving workloads were already high but further increased during the pandemic. Study 1 is based on semi‐structured interviews and its aim was to map the stressors that participants faced and understand their psychosocial impacts with a particular focus on identity‐related processes. Study 2 develops the qualitative findings by using survey data to measure the effects of both primary and secondary stressors on perceived stress, test the mediating effects of role overload and examine the effect of community identification on distress on top of the effects of stressors.

## STUDY 1

### Method

#### Participant recruitment

Our sample comprised 15 mothers of children and/or adolescents, interviewed between January and April 2021. During January 2021, England entered its third lockdown, whereas closer towards the end of February, a roadmap to lifting the lockdown was published. In March, primary and secondary schools reopened and towards the end of the month, outdoors gatherings of up to six people or two households were allowed and the ‘stay at home’ order ended. In April, non‐essential buildings and services reopened and so did outdoor venues.

Ethical approval was obtained by the Ethics Committee of Canterbury Christ Church University. Due to difficulties with recruitment caused by the pandemic, we used an opportunity sampling approach. Our only inclusion criterion was that participants should be mothers with at least one child under 16 years old at the time of the interviews. The second and third authors utilized existing nursery and school networks to approach participants. First, they approached potential participants known to them. Following the interviews, and using a snowballing technique, they asked participants to introduce them to other people in a similar situation with them, and many responded positively.

Initially, 17 mothers agreed to be interviewed. However, two participants withdrew their consent, leaving us with 15 interviews. All were White British. Three reported being stay‐at‐home mothers, four worked in education (e.g. researcher, lecturer, teacher), and two were administrators in education or medical clinics. One reported being a business owner and one self‐employed. For the remaining participants, the only information we received were that one was unemployed, two were working part‐time, and one was working full‐time. When we stopped observing new patterns in the data, we decided to not recruit further participants. Participant information is presented in Table 1.

**TABLE 1:** Participant characteristics.

Participant No.	Age range	No. of children	Children's ages at time of data collection	Partner	Co‐parent	Employment during COVID‐19
P1	40 – 50	2	11 and 14 years	Yes	No	Self‐employed (from home)
P2	40 – 50	2	13 and 18 years	Yes	Yes	Full‐time (from workplace)
P3	40 – 50	3	10,13 and 14 years	No	Yes	Full‐time (from home)
P4	40 – 50	2	10 and 13 years	Yes	No	Full‐time (from home)
P5	30 – 40	2	6 and 9 years	Yes	No	Stay at home mother
P6	30 – 40	2	4 months and 3 years	Yes	No	Part‐time (from home)
P7	40 – 50	2	3 and 10 years	Yes	No	Furloughed and self‐employed (from home)
P8	20 – 30	2	9 months and 3 years	Yes	No	Maternity/furloughed
P9	30 – 40	2	14 and 21 years	Yes	No	Full‐time (from home)
P10	40 – 50	2	11 and 13 years	Yes	No	Part‐time (in the workplace)
P11	40 – 50	2	7 and 10 years	Yes	No	Part‐time (from home)
P12	40 – 50	1	11 years	Yes	No	Stay‐at‐home mother
P13	40 – 50	2	7 and 11 years	Yes	No	Full‐time (from home)
P14	40 – 50	1	13 years	Yes	No	Self‐employed (from home)
P15	30 – 40	2	10 and 12 years	Yes	No	Furloughed and student

#### Interview schedule and study design

Qualitative data were collected by the second and third authors via semi‐structured interviews. Open‐ended questions were used, followed by specific prompts that encouraged participants to reflect on and provide narratives of their experiences, life conditions and stressors faced before, at the early onset and subsequent stages of the COVID‐19 pandemic. Interviews took place remotely, were recorded using Microsoft Teams and were transcribed verbatim, and the raw audio files were subsequently destroyed. All identifiable information was omitted during transcription; names and locations were removed to protect confidentiality. The interviews ranged between 40 and 70 min. The interview questions can be found here: https://osf.io/f94ce/?view_only=dc7d51a28a3442ea9c789ae7687398bb.

Prior to the commencement of the interviews, participants received a participant information sheet that included the purpose and requirements of the study, agreed to the interview being recorded and were asked to sign a consent form. Where written consent was not possible, it was provided verbally. Participants were informed that they could terminate the interview at any point and could have their data removed at any time by a specific date. Following completion of the interviews, participants were offered a debrief, detailing further information and support services should they feel they need to access help.

#### Procedure

To analyse our data, we used reflexive thematic analysis (Clarke et al., 2015). The second and third authors collected the data and transcribed them, with the first author checking the accuracy of the transcriptions. Next, the first three authors independently read through the dataset multiple times, taking notes on their initial observations. Our readings of the dataset were theoretically grounded on the concepts of secondary stressors, gender inequality and social identity processes in extreme events.

Subsequently, we compared our observations and used NVivo (https://lumivero.com/products/nvivo/) to create codes [or analytic units, Braun & Clarke, 2021]. Codes for example included sources of distress mentioned by participants or types of impacts. The team as a whole compared those codes, critically examined their interrelationships and developed themes structured around our aforementioned theoretical concerns. The quotes used to illustrate our points were selected based on their representativeness of the dataset as a whole. Also, because some stressors operated in multiple ways, in some cases we use multiple quotes to highlight this differentiation.

Participants appear by their respective number (e.g. P1 stands for ‘Participant 1). The symbol […] denotes text that has been removed to ease readability. Comments in [] are clarifications by the authors to provide additional context and add clarity.

### Results

We constructed six themes. Five themes capture different types of stressors and highlight their psychosocial impacts on participants, whereas the sixth theme describes coping mechanisms.

#### Theme 1: Feelings of captivity and overload due to a lack of partner support

Lack of partner support was commonly mentioned by participants:

P10: obviously the home, we had to home school. That was really tricky because I was trying to juggle both [work and homeschooling], and then my husband was working but then he did have actually three weeks off where he wasn't working because the sites shut. And I did ask him to help with home schooling, it didn't really happen. I was tearing my hair out slightly at that point.

P14: don't get me wrong, he's a brilliant dad but I do feel like it's all on me, like it's me checking the home schooling, and are they alright, making their food, and it's like having babies again, like it's that feeling that you can't get away, and that you're just like ‘oh god no, this whole house will collapse’.

Homeschooling was a new significant obligation that parents had to carry out during the pandemic. However, inequality was prevalent in terms of how such additional responsibilities were distributed among couples. For instance, P10 automatically positioned herself as trying to juggle both work and homeschooling, whereas her husband focused only on his work and did not engage with the additional tasks. Similarly, P14's disclaimer, which portrayed her husband as a good father (“don't get me wrong, he's a brilliant dad but…”), equally reveals that men were capable of retaining positive appraisals of their parenting identities despite not pulling their weight within the household. These data reveal how the institutionalized and gendered nature of family life operated during its interaction with an extreme event to overburden participants with additional responsibilities (“it's all on me…”). In some cases, participants did not receive adequate support from their partners even when there was capacity to do so (“he did have actually three weeks off”), creating a sense of regression to busy, past times (“like having babies again”).

Psychologically, the additional parenting tasks in conjunction with inequalities of task distribution within the household made participants feel overloaded and unable to escape their parenting identities (“that feeling that you can't get away”). Participants also reflected on how gender inequality shapes men's perceptions of their own privileged position within the household and of the role tasks expected to be taken up by women as part of their parenting identity:

P2: there seems to be an expectation that they are able to take themselves off to another room, shut the door and work a full office day, yeah when the other person is expected to teach and look after the children.

Participants also reported a sense of invisibility with regard to other aspects of their sense of self, which constrained them to their motherhood identity. For example, on top of lacking appropriate workspace at home, being overburdened with tasks and being constantly disrupted while trying to work, for P8's partner, her professional identity and responsibilities were not recognized:

P8: when I'm trying to work from home I have to sit in my bedroom and Wi‐Fi is rubbish and I'm having to be constantly interrupted by both children for both different needs and it's a nightmare and then my partner just thinks that I'm not actually working at home when I'm at home anyway.

#### Theme 2: homeschooling creating new obligations and distressing routines

In the previous theme, participants referred to lack of partner support in relation to a range of issues including homeschooling. However, homeschooling emerged as a separate stressor in itself. This is because, in order to reduce the spread of COVID‐19, schools closed and parents were obliged to manage their children's education by themselves at home, which affected them in multiple ways:

P10: There's concerns about certain subjects because she just could not get to grips with the online learning for certain subjects because the subject matter was just too difficult […] I've managed to help with certain subjects, but these, these are, they're beyond what I know. It was about the Cold War you know, I don't know anything about the Cold War, and I'd have to sit and research it but the, I'd think well this isn't right, that I've got to sit and research about the Cold War for a whole day

Many women's workload increased during lockdowns periods, partially facilitated by unequal task distribution within the household. As a result, educational policy shifts to homeschooling meant that the latter was more likely to fall on mothers rather than fathers and negatively affect them (Heers & Lipps, 2022). Considering that most people do not possess teaching or subject expertise but were suddenly required to do so, homeschooling proved to be a very time‐consuming activity that imposed an additional role in them, that of the teacher, and adding to their busy lives. However, homeschooling was not only a workload issue but also negatively impacted upon participants' quality of life:

P15: there was that dread of the maths or like that first morning bit of what kind of mood are they going to be in and even at breakfast and they say it's gonna be fine, the moment they get they could just change really quickly and then you've got that the battle for the morning is it just gonna ruin the day and then you're going to be arguing and at each other and you know there was the anxiety of how's it going to turnout you know

For many children, homeschooling affected their motivation to study and led to the loss of daily interactions with their friends and contact with their teachers. Some children's unwillingness or shifting attitudes towards homeschooling (“dread of maths”, “what kind of mood”, “could just change really quickly”) proved to be an additional burden for parents, and most likely for mothers, who now also had a teaching role to fulfil. Such daily hassles could be particularly problematic if persisting for long periods (cf. Norris & Uhl, 1993) and served to further constrain participants from other identities and identity‐related tasks apart from those related to parenting.

#### Theme 3: children's negative feelings and behaviours leading to exhaustion

Apart from homeschooling, parents were dealing with wider problematic behaviours that caused distress:

I: So have you had over the past 6 months any stressful experiences that you feel able to talk about?

P9: Any stressful experiences? I think I think my youngest one's anxiety, and it was getting horrendous and it started when she went back to school in September, it's quite noticeable um so that was quite tough actually, cos we've had to spend a lot of time working through things with her, um and comforting her and almost going back to basics again with her, like cos uh sleeping, she wasn't sleeping, like ‘it's alright you got anxiety about sleeping’ but she's getting almost obsessive so yeah that was quite tough actually.

Children's routines were also severely affected by the pandemic, which led to behavioural shifts. P5 stated that “lots of our kids went into crisis”, whereas P9 reflected on her daughter's shifting emotions and behaviours (“anxiety”, “wasn't sleeping”) because of returning to school. Overall, within the household, apart from taking on additional caring duties as well as the role of the teacher as demonstrated earlier, participants also had heightened concerns about their children's psychological well‐being but also had the additional task of coping and attempting to resolve such emerging issues, compounding the already existing difficulties. However, it was not only increased concerns about their children's psychological well‐being that were taxing for parents:

P8: I feel that actually she's missed out on a whole chunk of growing up and it's quite it's is quite showing with her because she does she's she's very immature at the minute yeah, and she's always always desperate for attention whenever I get home which is exhausting because I'm coming home from a full day's work and I'm exhausted. My partner walking in from like a 14 h day and he's exhausted and she's just bouncing about wanting attention and wants to do stuff, and can we go for a walk, and we can we play a game, and can we do this and now can I cook, now can I bake, and as much as that is kind of what you would do in a normal lifetime situation anyway it was never as intense as this

Due to rapid changes in their environments and routines, some children displayed new or heightened needs that parents had to address and experienced as an additional burden. In the case of P8, her daughter was described as developmentally “immature” due to the pandemic (“missed out on a whole chunk of growing up”) which led to increased needs for attention. This misalignment between parent's expectations of their children's behaviours and needs *vis a vis* the actual lived experience of constantly having to engage with their children proved to be an additional burden for parents who were already exhausted from other major obligations within and outside the household.

#### Theme 4: stress stemming from increased workload demands

Work‐related pressures were also taxing for parents in different ways:

P5: And so it all just felt like a bit relentless to be honest. Like there was… I was just desperately trying to think ‘how can I fit in my work…instead of vomiting’ (laughs) so yeah… that meant I was exhausted in the evenings and then straight switching immediately from doing your work to parenting instead of having any kind of wind down, or just any time to get into a different headspace.

P15: everyone's salary was down as well so there was definitely bit of tension kind of going on there and my manager was only around 4 days weeks instead of five and you know that was you definitely gotta feel that umm and in in the job […] work just got harder and harder and harder so I needed them to be at school really and my big leading up to leading up to Christmas so I had a really big project to start in January and my main thought was I cannot have the kids at home I cannot have in January I cannot do this, this is the biggest bit of my career ever, biggest project I've ever done and the most stressful.

The quotes presented above regarding the impacts of work‐related factors are telling. In some cases, like P5, having to simultaneously manage work duties and parenting was described as “relentless”. Reconciling the two was a difficult task, particularly for those participants who were forced to prioritize parenting over work. This would be increasingly difficult for participants without adequate partner support. The impact of these demands was clear—participants had no time for ‘winding down’ or getting into a ‘different headspace’. The significant work and parenting demands did not allow participants to be able to psychologically disengage from their parenting or work roles and identities, which were often salient simultaneously.

In other cases, such as P15, we were offered a glimpse into participants' institutional environments and how pandemic‐related changes in conjunction with intense parenting duties had a significant impact on participants' personal aspirations. P15's institution reduced full‐time working days from five to four, which led to reduced income and institutional support. P15 vividly described how her career aspirations were put at risk due to reductions in institutional support coupled with increased childcare duties. In other words, pandemic changes and a lack of adequate institutional support structures together with parenting duties that mostly fell on our female participants led to potentially tremendous impacts on other important self‐aspects that extended beyond the parenting identity (e.g. personal career aspirations).

#### Theme 5: the compound effects of stressors impacting on mothers' quality of life

A finding of theoretical interest was that the aforementioned stressors do not operate independently but rather their effects are compounded:

P9: I didn't have any time, I just had no time to myself at all because by the time I got up, done breakfast done a full day's work, homeschooled the kids, cooked and cleaned again and I was like ‘wow I'm too tired’ I'll sit down’, so there were days when I didn't leave the house, and it was like ‘well you gotta have time for yourself, well I better go and sit in the toilet’ you know what I mean? […] and it's like juggling 4 full plates at time, and I think I have come down the bottom of the pecking order if I'm honest.

The extract above indicates the compound effects of various stressors captured in the previous themes. The absence of personal time was frequently brought up by participants as a result, but one should further explicate the psychosocial dimensions of this issue. The women in our sample reported dealing with multiple tasks simultaneously (e.g. household tasks, childcare, homeschooling, work), a long‐lasting issue that made participants feel overwhelmed. There were also clear indications that household tasks were not being picked up by their partners which was contributing to the distress reported. The negative outcomes were multiple. Apart from the individual distress experienced, these women had no capacity to enjoy other parts of their social lives other than being carers for their households and children, and wished but could not avoid constantly being the centre of attention within the household. Some women resorted to locking themselves in the bathroom to regain some of their personal time, whereas others reported staying up late purposefully to experience some peace and quiet while their children were sleeping and to chat with their friends online. Nevertheless, this resulted in being tired the next day, ending up being an ongoing cycle of exhaustion and comprising an additional stressor in itself.

#### Theme 6: coping mechanisms: personal space and time, secure and supportive partner relationships, and enduring social connectedness and support groups

One individual coping strategy was to put effort in making time for oneself. Apart from P9 earlier who stated locking herself in the bathroom to regain some personal space, P8 would go for walks alone (“going for walks, without my daughter […] so actually going for the scans was kind of my time! It actually felt very nice!”). Environmental affordances such as having ample space both inside and outside at home also allowed participants to regain personal space and time from other household members.

P6: luckily because we do have the space in our house, we just sort of created two sort of office spaces that could be quite separate, which we're very fortunate and it meant that family life, had to be very kind of downstairs. That was ok because we do have the space, so we felt quite lucky about that.

High‐quality interpersonal relationships with their partners and more equal task division buffered negative experiences of stress and lessened participants' worries about their children and the household overall. P10 was hospitalized with COVID early in the pandemic. However, a strong dyadic relationship between P10 and her husband enabled the family to sustain positive coping mechanisms. Although P10 reported several health concerns, her husbands' support and the creation of a support bubble with her own mother, enabled them to cope.

P10: and while I was in hospital [my husband] sent me video of them out, he made up race tracks out the front and they were on their scooters doing races, the sun was out it was lovely um and he started baking, he did yeah so he'd instigated three o'clock snack times! […] I'm really lucky cos I've always always had a good relationship with my husband always, he's just always, he's always been there, it hasn't changed.

Whereas many mothers reported disconnecting from social media at times to protect themselves from news constantly perceived as negative, technology was beneficial. It helped participants sustain their social relationships with an extended community and harness the support and benefits they could gain from them such as experiencing a sense of common fate and togetherness due to facing similar stressors.

P2: We meet probably not that often probably once every 6 weeks on an online Zoom call and we have a proper catch up as girls and chat about everything… our struggles and about the kids and about our partners and about trying to juggle work and juggle home‐school and the kids just wanting to be stuck to a screen all day and not helping with the housework.

Collective coping also stemmed from actual support that originated in support ‘bubbles’, which were announced by the UK government as a method to see or use other people for support. Families tended to ‘buddy up’ with similar‐aged children, or grandparents, both for support and connection. When asked what would have made the biggest difference to her coping throughout the lockdowns P3 explained how the support bubbles were integral to how she coped (“If they put bubbles in in the first lockdown, bubbles have literally changed my life”).

## STUDY 2

Study 1 illustrated the complex pathways through which distress developed in mothers of children and/or adolescents at the onset of the pandemic, highlighting the significant psychosocial dimensions of secondary stressors. However, Study 1 left various questions unanswered, primarily due to its qualitative nature. For example, it could not disentangle whether secondary stressors were operating over and above concerns regarding the pandemic itself (the primary stressor), whether feeling captive or overloaded in terms of one's identity and position within the household could be a mediating mechanism between stressors and distress, or whether community identification, a group‐level variable, would be associated with distress over and above the effects of stressors. The latter is of particular interest considering that communities of different types (e.g. support bubbles with other families, online groups) were often reported as helpful at a time when the pandemic had largely constrained communal life at a physical level, forcing participants to largely operate within their households.

**FIGURE 1:**
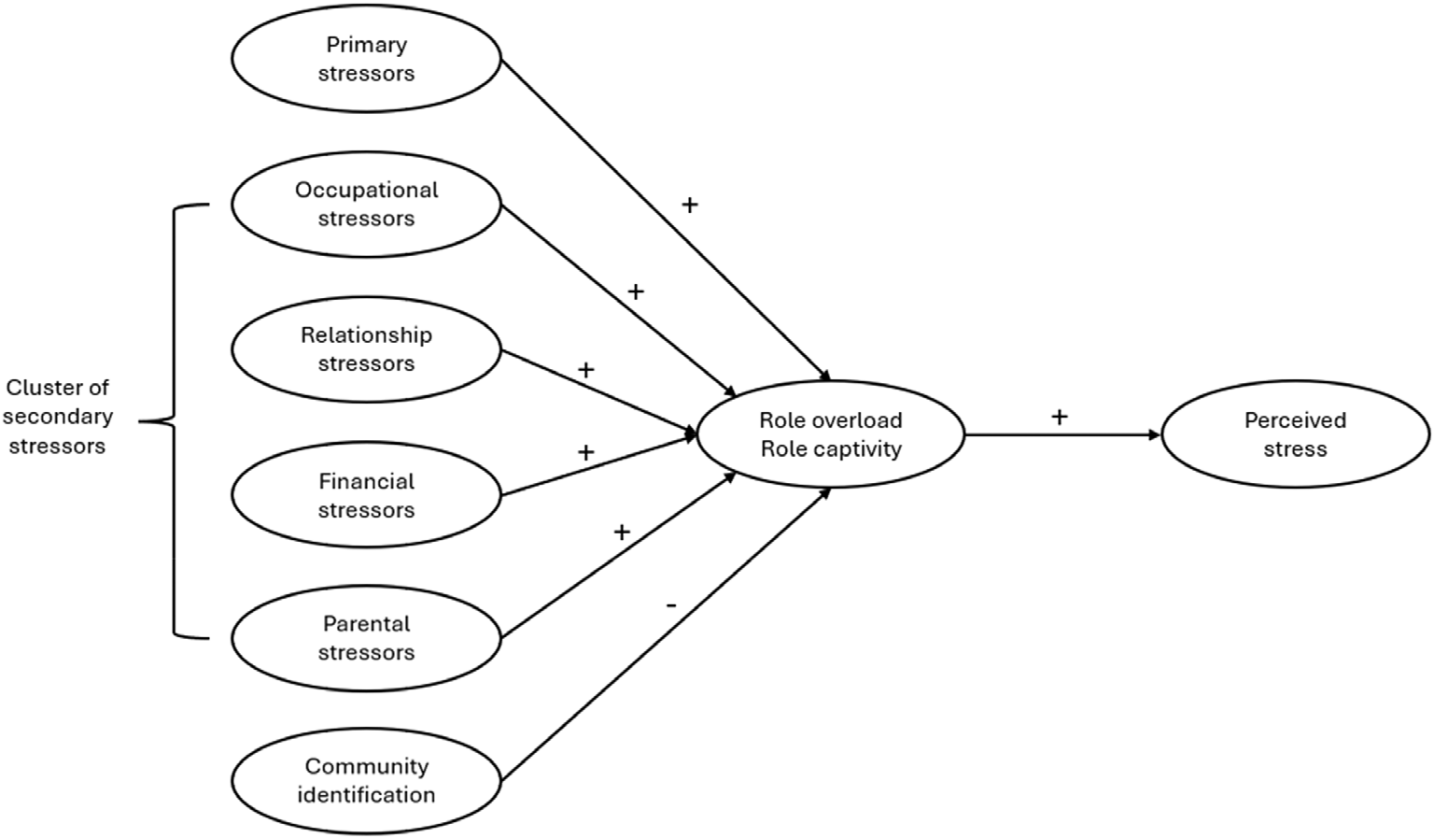
Diagram of tested model.

To answer these questions, we utilized survey data and hypothesized that primary stressors would be positively associated with perceived stress levels, and this relationship would be mediated by role overload and captivity (H1). Second, secondary stressors, acting together as a cluster, would be positively associated with perceived stress, and this relationship would be mediated by role overload and captivity (H2). Third, community identification would be negatively associated with perceived stress and this relationship would be mediated by role overload and captivity (H3). Figure 1 is a visual representation of our hypotheses.

### Method

#### Participants and procedure

The survey study was conducted between August 17 and 19, 2021, a month after most legal limits on social contact were lifted in the United Kingdom and a month before all the schools reopened without restrictions. Data were collected during a transition period in which most people and children resumed their ordinary lives and daily activities after being under several restrictions because of the pandemic.

Prior to data collection, we conducted an a priori power analysis employing Monte Carlo simulations for structural equation models through the software pwdSEM (see Wang & Rhemtulla, 2021). The sample size required to calculate the tested model (see Figure 1) was determined considering five indicators for each latent variable; high factor loadings (*λ* = .75); small effect size from the independent variables to the mediator and from the mediator to the dependent variable (*d* = 0.35); low direct effects from the independent variables to the dependent variable (*d* = 0.2); and low covariances among the independent variables (*r* = .10). Conducted simulations indicated that the minimum sample size to achieve 85% power and a significance criterion of *α* = .05 was *N* = 400.

Ethical approval was issued by the Ethics Committee of [ANONYMIZED FOR REVIEW] University. All participants (*N* = 621; *M*_age_ = 33.78, *SD*_age_ = 5.35) were recruited through Prolific, receiving £1.85 each after completing a 15 min online survey. Our sample comprises mothers from England (82.1%), Scotland (7.5%), Wales (4.5%) and Northern Ireland (2.2%). Most of the surveyed mothers (89.7%) had one or two children under the age of 12 at home, while most were either full or part‐time paid employees (64.9%). A significant proportion of participants declared being married (54.9%) or in a relationship (32.3%), with a few being single mothers (9.5%) or divorced (3.2%). Regarding participants' educational background, 57% had completed higher education at different levels (degree, master, or PhD). The survey was not pre‐registered.

#### Measures

##### Primary stressors

Mothers' concerns regarding issues directly related to the pandemic were measured through five items (*⍵* = .87) describing situations where either participants' health or the life of significant ones were threatened by coronavirus (e.g. ‘T*he possibility of my children and other family members contracting COVID‐19’*). Each item was assessed using a scale ranging from 1 ‘Not at all’ to 5 ‘Completely’. Items were adapted from previous research that had measured what theoretically is classified as a primary stressor (e.g. Blanco‐Donoso et al., 2021; Darlington et al., 2021).

##### Secondary stressors

Participants reported their levels of concern about various secondary stressors during the 6 months prior to data collection on a scale ranging from 1 ‘Never’ to 5 ‘Very often’. A set of five items (*⍵* = .90) measured occupational concerns caused by the impact of the pandemic on job‐related issues (e.g. ‘*Were you treated unfairly by others in the job*?’); five items (*⍵* = .88) were aimed at exploring issues related to relationship struggles (e.g. ‘*Did you feel that your partner was not contributing equally to household tasks (e.g. home‐schooling, housework)?*’); four items (*⍵* = .85) were used to measure mothers' financial concerns during the pandemic (e.g. *‘Did you have trouble meeting the monthly payments on bills?*’); while four items (*⍵* = .88) measured sources of stress associated with parental duties (‘*Were you concerned about your children's mental health?*’). These items were identified in previous work in this field (Norris & Uhl, 1993; Pearlin et al., 1990; Tempest et al., 2017) and were adapted to reflect the particular pandemic context.

##### Role overload

Participants reported the extent of being overloaded by multiple demands through four items (*⍵* = .83) (e.g. ‘*You have more things to do than you can handle*’). Each statement was ranked by participants using a scale of measurement ranging from 1 ‘Not at all’ to 5 ‘Completely’. Items were adapted from Pearlin et al. (1990).

##### Role captivity

Three items were employed to report participants' sense of captivity stemming from their maternal duties (*⍵* = .81) (e.g. ‘*You feel trapped by your childcare responsibilities*’). Each statement was ranked by participants using a scale of measurement ranging from 1 ‘Not at all’ to 5 ‘Completely’. Items were adapted from Pearlin et al. (1990).

##### Community identification

Mothers' identification with their local communities was measured through three items (e.g. *‘I have a sense of belonging to my local community’*) using a scale ranging from 1 ‘Strongly disagree’ to 5 ‘Strongly agree’ (*⍵* = .91). Items were adapted from Sani et al. (2015).

##### Perceived stress

Participants were asked to indicate their stress levels through five items (*⍵* = .86) representing diverse daily‐life situations. For each item, participants reported the frequency of being ‘upset’ ‘nervous’ or ‘angered’ in the 6 months before data collection. Participants rated each affirmation (e.g. ‘*in the last six month how often have you been upset because something happened unexpectedly*’) on a scale ranging from 1 ‘Never’ to 5 ‘Very often’. Items were adapted from Cohen et al.'s (1983) Perceived Stress Scale.

##### Demographics

Participants reported their country of residence (i.e. England, Scotland, Wales, Northern Ireland), age, income, number of children under 12 at home, work status (e.g. full‐time paid employee) and relationship status (i.e. single, married, divorced, widow).

Originally, the survey included more scales and more items in each respective scale.^1^ The items used in the analysis (and reported earlier in this section) are those that were deemed the most appropriate following a Confirmatory Factor Analysis. The survey items, raw data, analysis code and codebook can be found here: https://osf.io/f94ce/?view_only=dc7d51a28a3442ea9c789ae7687398bb. A correlation matrix appears in Table 2.

**TABLE 2:** Correlations among latent variables Study 2.

	1	2	3	4	5	6	7	8	9
1. Primary stressors	–								
2. Community identification	.05	–							
3. Occupational stressors	.12*	.00	–						
4. Relationship stressors	.09*	−.09	.19***	–					
5. Financial stressors	.10*	−.14*	.14***	.21***	–				
6. Parental stressors	.17***	−.01	.14**	.22**	.25***	–			
7. Role overload	.31***	−.21**	.28***	.52***	.29***	.25***	–		
8. Role captivity	−.01	−.20***	.23***	.41***	.25***	.25***	.59***	–	
9. Perceived stress	.21***	−.23***	.28***	.45***	.27***	.28***	.71***	.56***	–

*p < .05.

**p < .01.

***p < .001.

#### Results

The first stage of the analysis explored whether demographic variables exerted any effects on perceived stress, the main dependent variable of this model. We conducted a series of ANOVAs using a composite measure of items from the perceived stress scale. Perceived stress levels did not vary based on income (*F*(11,604) = 0.95, *p* = .48); number of children at home under the age of 12 (*F*(3,612) = 0.15, *p* = .93); work status (i.e. working mothers vs. non‐working mothers) (*F*(1,613) = 2.72, *p* = .09); relationship status (*F*(3,612) = 0.34, *p* = .79); or country of residence (*F*(4,611) = 1.44, *p* = .21).

For the second stage of analysis, we used structural equation modelling with latent variables and maximum likelihood robust (MLR) as estimator to test the proposed theoretical model and the hypotheses driving this study. MLR allowed us to calculate our model assuming non‐continuous data and missing completely at random (MCAR). Thus, we were able to conduct our analysis without excluding observations with missing data.

Due to the high correlation between role overload and captivity, we opted to use only one of the two variables when testing our model. Additionally, demographic variables exhibited no correlations with the key variables, therefore we did not include them as covariates.

Overall, the proposed independent variables (stressors, community identification) and the mediator (role overload) correlated highly with perceived stress. In contrast, independent variables (i.e. primary stressors, secondary stressors and community identification) demonstrated low correlations between them.

**FIGURE 2:**
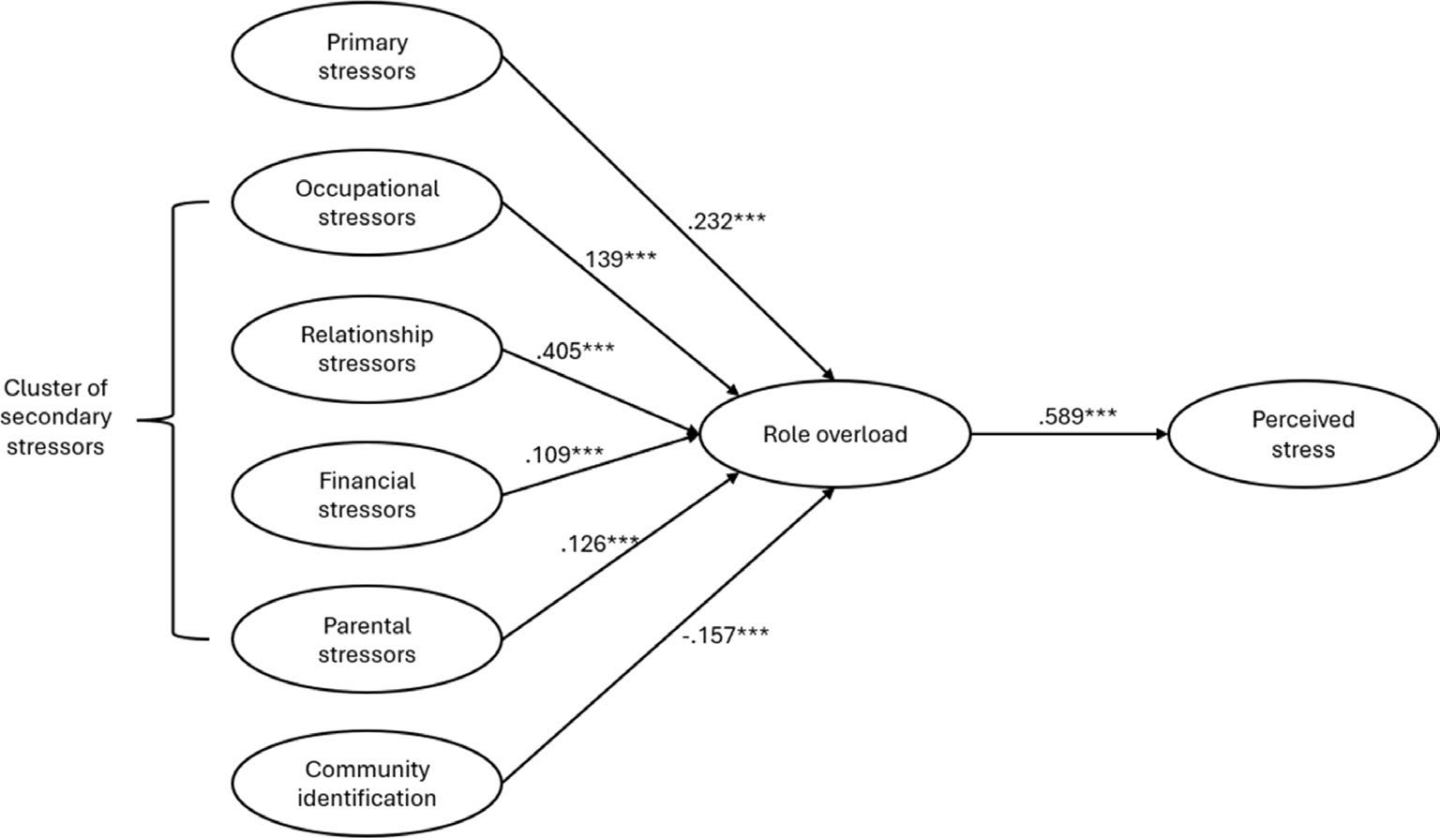
Model diagram of survey analysis results. ***p < .001.

The tested model demonstrated an excellent fit to the data (*χ* ^*2*^ (532) = 980.71; *p* < .001; *CFI* = .96; *TLI* = .95; *RMSEA* = .03; *SRMR* = .04), explaining 53% of the variance of perceived stress (see Figure 2). Primary stressors had a significant impact on perceived stress through role overload (*β* = .13, *SE* = .02, *z* = 5.14, *p* < .001), supporting H1.

Secondary stressors had a direct impact on perceived stress through increasing feelings of role overload, supporting H2. More specifically, the effects of secondary stressors on perceived stress were either partially or fully mediated by role overload. In particular, the impact of occupational (*β* = .08, *SE* = .02, *z* = 3.37, *p* < .001) and relational stressors (*β* = .23, *SE* = .03, *z* = 7.19, *p* < .001) on perceived stress was partially mediated via role overload. Moreover, overload fully mediated the effects of financial concerns (*β* = .06, *SE* = .02, *z* = 2.41, *p* < .001) and parental duties (*β* = .07, *SE* = .02, *z* = 2.66, *p* = .008) on perceived stress. It is worth noting that those situations related to relationship struggles might be more problematic for mothers (*β* = .40, *SE* = .04, *z* = 9.65, *p* < .001) and therefore considered a more critical source of role overload feelings by our participants in comparison to those issues associated with mothers' workload, financial situation and parental duties.

H3 was also supported. Community identification was associated with reduced perceived stress and this relationship was mediated by reduced overload (H3; *β* = −.09, *SE* = .02, *z* = −3.59, *p* < .001).

## GENERAL DISCUSSION

This paper adopted a mixed‐methods design to explore the psychosocial processes through which stressors exerted their negative influence on mothers of children and/or adolescents in the United Kingdom during the COVID‐19 pandemic. Our emphasis on secondary stressors is in line with Erikson and Peek's (2022) argument that apart from their main impact, extreme events serve as a lens to reveal a range of problems that precede them and unfortunately magnify their effects. This points towards the need for researchers to contextualize the impact of extreme events by examining in greater detail the social environments and networks within which they manifest and people's histories and experiences of them.

In our case, interview data illustrated the complex and interrelated pathways through which the stress process operated during the earlier stages of the pandemic. A lack of partner support left many participants feeling overwhelmed and unable to escape from their duties, roles and identities. Participants identified role‐based expectations in their relationships, exasperated by stress and contributing to the sense of captivity. This sense of captivity was compounded by the additional burden of homeschooling, which many mothers were unprepared for, leading to heightened stress and anxiety. Children's behavioural problems further exacerbated distress due to increasing exhaustion. Increased workload demands, failing career aspirations and additional children's needs in terms of practical and emotional support further stretched and exhausted parents and at the same time prevented our participants from exercising other significant aspects of their social selves. Crucially, secondary stressors operated simultaneously despite spanning across multiple domains (e.g. relationships, workplaces, household), exacerbating tensions in the family system and within individuals. However, enduring social connectedness, supportive partners and material affordances were referred to as protective factors.

Survey data showed that participants' stress was triggered by both primary and secondary stressors, with the latter (e.g. job demands, relationship struggles, financial concerns, parental duties) playing a leading role. The relationship between stressors and perceived stress was mediated by role overload, highlighting the negative influence of particular social positions and arrangements (i.e. being a woman/mother within a context of gender inequality) on mental well‐being. In line with the qualitative findings, secondary stressors operated as a cluster and exerted independent but simultaneous effects on participants. Community identification however exerted independent beneficial effects, reducing perceived stress via lower levels of overload.

Our studies speak to a range of different issues in the domains of social sciences in general and in social psychology regarding studying extreme events. First, at risk of overgeneralising, social scientific research on distress in extreme events has mainly endorsed one of two approaches. On the one hand, there has been a focus on social determinants of health—macro‐level variables such as income, gender and health inequalities that can affect physical and mental health (Davidson, 2014). On the other hand, an epidemiological lens has explored the relationship between stressors and the prevalence of mental health disorders (e.g. Jermacane et al., 2018; Mulchandani et al., 2019; Munro et al., 2017). However, the former, despite identifying impactful variables of interest, does not highlight the microprocesses through which macro‐level problems (e.g. gender inequality) become potent within particular social environments for particular groups. The latter, despite identifying relationships between stressors and outcomes, often operates descriptively without highlighting the psychosocial processes through which stressors appear and exert their negative influences.

Our theoretical interest in the social and institutional dimensions of stress and trauma, also informed by recent developments in the social identity processes of health and identity change, addresses the aforementioned literature gaps. This is achieved by outlining how macro‐level inequalities (e.g. regarding gender) translate into specific stressors during extreme events that shape people's routines and their relationships with other members in particular environments, eventually constraining various aspects of their social selves and becoming distressing. We showed that, when shaped by gender inequality, the family system can be a burdensome environment for mothers due to a multitude of stressors that entrap them into particular social identities and constrain alternatives. Our analysis also highlights the benefits of utilizing both qualitative and quantitative methods as these allow us to understand the texture of participants' experiences and lives, isolate variables of theoretical interest and translate them into statistical models that test particular predictions in wider populations. This type of analysis can make significant contributions towards a systems‐thinking approach to exploring the psychological impacts of extreme events, which requires a synergy of theoretical approaches and analytic skills to examine how diverse factors interact at different levels to produce outcomes of interest (see Berry et al., 2018).

Second, our paper contributes to existing social psychological research on social identity processes and their relation to health and stress (Haslam et al., 2018; Haslam & Reicher, 2006; Wakefield et al., 2019), trauma (Muldoon, 2024) and identity change (Haslam et al., 2021; Muldoon, 2024; Muldoon et al., 2019). This strand of work has been applied to extreme events to examine, among others, the emergence of groups and solidarity (Drury et al., 2019; Ntontis et al., 2019; Ntontis, Drury, Amlot, et al., 2020; Ntontis, Drury, Amlôt, et al., 2020), post‐traumatic stress (Muldoon et al., 2017) and resilience and recovery (Stancombe et al., 2023), highlighting the benefits incurred by a strong sense of psychological connectedness to social groups. However, social groups, depending on their constituting norms and identity content, can also be a source of harm and distress (Kellezi, 2012; Kellezi et al., 2021; Wakefield et al., 2019).

Our findings point towards both processes. On the one hand, our data showed that social connectedness, supportive partnerships and community identification served to make participants feel supported and reduce the sense of captivity and distress. On the other hand, the pandemic caused shifts in participants' environments and routines by constraining social life within the household and limiting physical interactions with other people. The existing literature has highlighted how periods of crisis and transitions can cause identity change or loss, undermining people's sense of self and their networks and availability of social support (Haslam et al., 2021; Muldoon, 2024). Moreover, it has investigated the contextual factors that allow or prevent people to enact valued identities, which can be beneficial for well‐being (Hopkins et al., 2023). Our studies highlight a different traumatic pattern; not one of participants losing or not being able to practise some valued identities, but one of not being able to escape from particular identities, overwhelming obligations and systems of relations, and not being able to exercise other valued aspects of their social selves. The sense of entrapment and overload stemming from inequality can also affect how those affected appraise both the stressors and their capacity to cope (see Lazarus & Folkman, 1984). More generally, systems rife with inequality which also prescribe specific identity‐based tasks to specific members can increase strains and negative outcomes (e.g. feelings of captivity or overload), rendering the social identity problematic but at the same time often not possible to let go of. Thus, whereas in some cases some social systems can become sources of coping, in other cases they can challenge the capacity to cope, especially in extreme events like a pandemic where mobility and other sources of coping (e.g. extended family, friends and other social groups) are impossible to reach due to external restrictions.

Third, an emerging literature focuses on complex disasters with protracted, cascading and compounded effects (Cutter, 2018; Kruczkiewicz et al., 2021; Lukasiewicz & O'Donnell, 2022) at a time when the climate crisis is increasing the prevalence of extreme events in a society that is already rife with inequality and inequity. In our case, we focused on a single event, the COVID‐19 pandemic, which nevertheless was a long‐duration hazard, with multiple pandemic waves and an ever‐present primary stressor that over time became less of a major threat. The persisting presence of the hazard can cause latent forms of vulnerability to arise, which are outcomes of and exacerbated by pre‐existing patterns of inequality. New routines in people's social lives are created as forms of adaptation to the new reality (i.e. the long‐term presence of COVID‐19) which might disadvantage particular social actors. In other words, our participants were probably being exposed to forms of inequality even before the pandemic due to their disadvantaged status as women and mothers in a society rife with gender‐based discrimination. However, the hazard created new pathways towards being disadvantaged and as a corollary, novel long‐term psychosocial impacts fuelled by particular social identities and ascribed roles and obligations. It becomes mandatory then for disaster risk management to consider the interaction between hazards and the (unequal) systems they affect over time and to consider the compounding effects of the hazard and pre‐disaster and emerging inequalities on people (Few et al., 2020; Lukasiewicz & O'Donnell, 2022). This becomes paramount if we consider the increasingly complex impacts of disasters on both individuals and communities which are more interconnected than ever, leading to a complex web of material and psychosocial impacts. Our analysis brought to surface a range of compounded secondary stressors that operated as clusters in only one event. Considering that multiple interacting extreme events can interact with systemic inequalities which carry their own clusters of secondary stressors, then multidisciplinary approaches using diverse methods across different levels of analysis will be of central importance in the future.

### Limitations and future research

Our studies suffer from a range of limitations. First, our sample comprises predominantly white, middle‐class women in the United Kingdom. Most of our participants in the qualitative study lived in large houses in England, had one or two children, did not have very young children and had adequate indoor and outdoor space to readjust their lives during the pandemic. Future research should explore how these processes manifest in mothers who are not White, are less financially affluent, might have younger children or reside in societies with differing gender norms, roles and caregiving responsibilities. This is because some people might be less affluent and live in more crowded spaces which can intensify conflict, might live in less affluent countries in general, might live in multi‐generational households or might have more children. Alternatively, much younger children (e.g. babies, young toddlers) might create additional or different needs that we could not capture. Our survey data also captured the number of children under 12 years old, but we did not include items to tease these numbers apart, which could yield useful insights based on children's developmental stages. In all these cases, resources might be few, but needs can be high, or stressors might be different compared to what we described earlier, posing different challenges. Thus, our study does not represent the experiences of all mothers and more research with different population across different cultures is required.

Future research can also explore participants' appraisals of their partners or other groups as coping resources and measure different types of social identifications and examine the availability and perceptions of social support. Finally, future research should utilize a longitudinal design to explore how the effects of stressors change over time and how they can be modified by psychosocial resources at different timepoints.

## CONCLUSION

In this paper, we used a social identity framework and set out to examine the psychosocial processes through which stressors become traumatic for mothers of children and/or adolescents during extreme events. Drawing on a combination of qualitative and quantitative methods, we mapped the stressors that participants faced and examined how experiences of distress were influenced by impacts on participants' sense of self. Our analysis reaffirms the need for (social) psychology to engage seriously with social structures and institutional arrangements and how these exert negative psychosocial impacts on people's life‐worlds.

## AUTHOR CONTRIBUTIONS

**Patricio Saavedra:** Conceptualization; investigation; methodology; validation; visualization; writing – review and editing; software; formal analysis; data curation. **Aida Malovic:** Supervision; conceptualization; writing – review and editing; validation; formal analysis. **Jennifer Monkhouse:** Writing – review and editing; formal analysis; data curation; investigation. **Natalie Stokes‐Guizani:** Investigation; writing – review and editing; data curation; formal analysis. **Evangelos Ntontis:** Conceptualization; writing – original draft; funding acquisition; investigation; methodology; validation; visualization; writing – review and editing; formal analysis; project administration; supervision; data curation.

## FUNDING INFORMATION

The survey study was funded through a QR grant awarded by Canterbury Christ Church University to Evangelos Ntontis.

## CONFLICT OF INTEREST STATEMENT

The authors declare that there are no potential conflicts of interest with respect to the research, authorship and/or publication of this article.

## ETHICS STATEMENT

Ethical approval for the studies reported below was acquired by the Ethics Committee of Canterbury Christ Church University before the studies commenced.

## LINK TO MATERIALS AND CODE

https://osf.io/f94ce/?view_only=dc7d51a28a3442ea9c789ae7687398bb

## Data Availability

The data that support the findings of this study are openly available in OSF at https://osf.io/f94ce/.

## Footnotes

^1^
We originally used more scales such as on well‐being and perceived and received social support. However, a factor analysis showed that they did not scale as intended as thus we could not use them in our analyses (e.g. to see how social support can also operate as a stress buffer).

## References

[bjso12856-bib-0001] Almeida, M. , Shrestha, A. D. , Stojanac, D. , & Miller, L. J. (2020). The impact of the COVID‐19 pandemic on women's mental health. Archives of Women's Mental Health, 23, 741–748. 10.1007/s00737-020-01092-2 PMC7707813 33263142

[bjso12856-bib-0002] Arntz, M. , Ben Yahmed, S. , & Berlingieri, F. (2020). Working from home and COVID‐19: The chances and risks for gender gaps. Intereconomics, 55, 381–386. 10.1007/s10272-020-0938-5 33281218 PMC7704591

[bjso12856-bib-0003] Benin, M. , & Keith, V. M. (1995). The social support of employed African American and Anglo mothers. Journal of Family Issues, 16(3), 275–297. 10.1177/019251395016003003

[bjso12856-bib-0004] Berry, H. L. , Waite, T. D. , Dear, K. B. G. , Capon, A. G. , & Murray, V. (2018). The case for systems thinking about climate change and mental health. Nature Climate Change, 8(4), 282–290. 10.1038/s41558-018-0102-4

[bjso12856-bib-0005] Blanco‐Donoso, L. M. , Moreno‐Jiménez, J. , Amutio, A. , Gallego‐Alberto, L. , Moreno‐Jiménez, B. , & Garrosa, E. (2021). Stressors, job resources, fear of contagion, and secondary traumatic stress among nursing home workers in face of the COVID‐19: The case of Spain. Journal of Applied Gerontology, 40(3), 244–256. 10.1177/0733464820964153 33025850

[bjso12856-bib-0006] Braun, V. , & Clarke, V. (2021). One size fits all? What counts as quality practice in (reflexive) thematic analysis? Qualitative Research in Psychology, 18(3), 328–352. 10.1080/14780887.2020.1769238

[bjso12856-bib-0007] Burki, T. (2020). The indirect impact of COVID‐19 on women. The Lancet Infectious Diseases, 20(8), 904–905. 32738239 10.1016/S1473-3099(20)30568-5 PMC7836874

[bjso12856-bib-0008] Clarke, V. , Braun, V. , & Hayfield, N. (2015). Using thematic analysis in psychology. In J. A. Smith (Ed.), Qualitative psychology: A practical guide to research methods. Sage.

[bjso12856-bib-0009] Cohen, S. , Kamarck, T. , & Mermelstein, R. (1983). A global measure of perceived stress. Journal of Health and Social Behavior, 24(4), 386–396. 10.2307/2136404 6668417

[bjso12856-bib-0010] Cutter, S. L. (2018). Compound, cascading, or complex disasters: What's in a name? Environment: Science and Policy for Sustainable Development, 60(6), 16–25. 10.1080/00139157.2018.1517518

[bjso12856-bib-0011] Darlington, A. S. E. , Morgan, J. E. , Wagland, R. , Sodergren, S. C. , Culliford, D. , Gamble, A. , & Phillips, B. (2021). COVID‐19 and children with cancer: Parents' experiences, anxieties and support needs. Pediatric Blood & Cancer, 68(2), e28790. 10.1002/pbc.28790 33219739 PMC7744834

[bjso12856-bib-0012] Davidson, A. (2014). Social determinants of health: A comparative approach. Oxford University Press.

[bjso12856-bib-0013] Demirtepe‐Saygılı, D. , & Bozo, Ö. (2010). Predicting depressive symptoms among the mothers of children with leukaemia: A caregiver stress model perspective. Psychology & Health, 26(5), 585–599. 10.1080/08870441003611577 21038170

[bjso12856-bib-0014] Department of Health . (2009). NHS emergency planning guidance: Planning for the psychosocial and mental health Care of People Affected by major incidents and disasters: Interim National Strategic Guidance. The Department of Health. https://www.coe.int/t/dg4/majorhazards/ressources/virtuallibrary/materials/UK/dh_103563.pdf

[bjso12856-bib-0015] Drury, J. , Brown, R. , González, R. , & Miranda, D. (2016). Emergent social identity and observing social support predict social support provided by survivors in a disaster: Solidarity in the 2010 Chile earthquake. European Journal of Social Psychology, 46(2), 209–223. 10.1002/ejsp.2146

[bjso12856-bib-0016] Drury, J. , Carter, H. , Cocking, C. , Ntontis, E. , Guven, S. T. , & Amlot, R. (2019). Facilitating collective resilience in the public in emergencies: Twelve recommendations based on the social identity approach. Frontiers in Public Health, 7, 1–21. 10.3389/fpubh.2019.00141 31214561 PMC6558061

[bjso12856-bib-0017] Erikson, K. (1976). Everything in its path: Destruction of Buffalo Creek. Simon & Schuster.

[bjso12856-bib-0018] Erikson, K. (1994). A new species of trouble: Explorations in disaster, trauma and community. W. W. Norton & Company.

[bjso12856-bib-0019] Erikson, K. , & Peek, L. (2022). The continuing storm: Learning from Katrina. University of Texas Press.

[bjso12856-bib-0020] Few, R. , Chhotray, V. , Tebboth, M. , Forster, J. , White, C. , Armijos, T. , & Shelton, C. (2020). COVID‐19 crisis: Lessons for recovery. In What can we learn from existing research on the long‐term aspects of disaster risk and recovery? The British Academy. https://www.thebritishacademy.ac.uk/publications/covid‐19‐crisis‐lessons‐recovery/

[bjso12856-bib-0021] Goldin, C. (2022). Understanding the economic impact of COVID‐19 on women. Brookings Papers on Economic Activity, 2022, 65–139.

[bjso12856-bib-0022] Goldmann, E. , & Galea, S. (2014). Mental health consequences of disasters. Annual Review of Public Health, 35(1), 169–183. 10.1146/annurev-publhealth-032013-182435 24159920

[bjso12856-bib-0023] Haslam, C. , Haslam, S. A. , Jetten, J. , Cruwys, T. , & Steffens, N. K. (2021). Life change, social identity, and health. Annual Review of Psychology, 72(1), 635–661. 10.1146/annurev-psych-060120-111721 32886584

[bjso12856-bib-0024] Haslam, C. , Jetten, J. , Cruwys, T. , Dingle, G. A. , & Haslam, S. A. (2018). The new psychology of health: Unlocking the social cure (1st ed.). Routledge.

[bjso12856-bib-0026] Haslam, S. A. , O'Brien, A. , Jetten, J. , Vormedal, K. , & Penna, S. (2005). Taking the strain: Social identity, social support, and the experience of stress. British Journal of Social Psychology, 44(3), 355–370. 10.1348/014466605X37468 16238844

[bjso12856-bib-0027] Haslam, S. A. , & Reicher, S. (2006). Stressing the group: Social identity and the unfolding dynamics of responses to stress. Journal of Applied Psychology, 91(5), 1037–1052. 10.1037/0021-9010.91.5.1037 16953766

[bjso12856-bib-0028] Heers, M. , & Lipps, O. (2022). Overwhelmed by learning in lockdown: Effects of COVID‐19‐enforced homeschooling on parents' wellbeing. Social Indicators Research, 164(1), 323–343. 10.1007/s11205-022-02936-3 35761906 PMC9218707

[bjso12856-bib-0029] Hopkins, N. , Ryan, C. , Portice, J. , Straßburger, V. M. , Ahluwalia‐McMeddes, A. , Dobai, A. , Perhson, S. , & Reicher, S. (2023). Social identity enactment in a pandemic: Scottish Muslims' experiences of restricted access to communal spaces. British Journal of Social Psychology, 62(3), 1141–1157. 10.1111/bjso.12625 36715002

[bjso12856-bib-0030] Jermacane, D. , Waite, T. D. , Beck, C. R. , Bone, A. , Amlôt, R. , Reacher, M. , Kovats, S. , Armstrong, B. , Leonardi, G. , Rubin, G. J. , & Oliver, I. (2018). The English National Cohort Study of flooding and health: The change in the prevalence of psychological morbidity at year two. BMC Public Health, 18, 1–8. 10.1186/s12889-018-5236-9 PMC5842606 29514665

[bjso12856-bib-0031] Jetten, J. , Haslam, S. A. , Iyer, A. , & Haslam, C. (2009). Turning to others in times of change: Social identity and coping with stress. In S. Stürmer & M. Snyder (Eds.), The psychology of prosocial behavior: Group processes, intergroup relations, and helping (pp. 139–156). Blackwell. 10.1002/9781444307948.ch7

[bjso12856-bib-0032] Kaniasty, K. , & Norris, F. (1999). The experience of disaster: Individuals and communities sharing trauma. In R. Gist & B. Lubin (Eds.), Response to disaster: Psychosocial, community, and ecological approaches (pp. 25–62). Bruner/Mazel.

[bjso12856-bib-0033] Kaniasty, K. , & Norris, F. H. (1995). In search of altruistic community: Patterns of social support mobilization following hurricane Hugo. American Journal of Community Psychology, 23(4), 447–477. 10.1007/BF02506964 8546107

[bjso12856-bib-0034] Kellezi, B. (2012). Social cure or social curse?: The psychological impact of extreme events during the Kosovo conflict. In J. Jetten , C. Haslam , & A. Haslam (Eds.), The social cure: Identity, health and wellbeing (pp. 217–233). Psychology Press.

[bjso12856-bib-0035] Kellezi, B. , Guxholli, A. , Stevenson, C. , Ruth Helen Wakefield, J. , Bowe, M. , & Bridger, K. (2021). ‘Enemy of the people’: Family identity as social cure and curse dynamics in contexts of human rights violations. European Journal of Social Psychology, 51(3), 450–466. 10.1002/ejsp.2750

[bjso12856-bib-0036] Kruczkiewicz, A. , Klopp, J. , Fisher, J. , Mason, S. , McClain, S. , Sheekh, N. M. , Moss, R. , Parks, R. M. , & Braneon, C. (2021). Compound risks and complex emergencies require new approaches to preparedness. Proceedings of the National Academy of Sciences of the United States of America, 118(19), e2106795118. 10.1073/pnas.2106795118 33952695 PMC8126784

[bjso12856-bib-0037] Lachance‐Grzela, M. , & Bouchard, G. (2010). Why do women do the lion's share of housework? A decade of research. Sex Roles, 63, 767–780. 10.1007/s11199-010-9797-z

[bjso12856-bib-0038] Lazarus, R. S. , & Folkman, S. (1984). Stress, appraisal, and coping. Springer publishing company.

[bjso12856-bib-0039] Lease, S. H. (1999). Occupational role stressors, coping, support and hardiness as predictors of strain in academic faculty: An emphasis on new and female faculty. Research in Higher Education, 40, 285–307. 10.1023/A:1018747000082

[bjso12856-bib-0040] Lukasiewicz, A. , & O'Donnell, T. (2022). The evolution of complex disasters. In A. Lukasiewicz & T. O'Donnell (Eds.), Complex disasters (pp. 3–19). Springer Nature. 10.1007/978-981-19-2428-6_1

[bjso12856-bib-0041] Medina, S. , & Magnuson, S. (2009). Motherhood in the 21st century: Implications for counselors. Journal of Counseling & Development, 87(1), 90–96. 10.1002/j.1556-6678.2009.tb00553.x

[bjso12856-bib-0042] Mulchandani, R. , Smith, M. , Armstrong, B. , Beck, C. R. , Oliver, I. , Davidwaite, T. , Bone, A. , Amlôt, R. , Kovats, S. , Leonardi, G. , & James Rubin, G. (2019). Effect of insurance‐related factors on the association between flooding and mental health outcomes. International Journal of Environmental Research and Public Health, 16(7), 1–9. 10.3390/ijerph16071174 PMC6480571 30986906

[bjso12856-bib-0043] Muldoon, O. T. (2024). The social psychology of trauma: Connecting the personal and the political. Cambridge University Press.

[bjso12856-bib-0044] Muldoon, O. T. , Acharya, K. , Jay, S. , Adhikari, K. , Pettigrew, J. , & Lowe, R. D. (2017). Community identity and collective efficacy: A social cure for traumatic stress in post‐earthquake Nepal. European Journal of Social Psychology, 47(7), 904–915. 10.1002/ejsp.2330

[bjso12856-bib-0045] Muldoon, O. T. , Haslam, S. A. , Haslam, C. , Cruwys, T. , Kearns, M. , & Jetten, J. (2019). The social psychology of responses to trauma: Social identity pathways associated with divergent traumatic responses. European Review of Social Psychology, 30(1), 311–348. 10.1080/10463283.2020.1711628

[bjso12856-bib-0046] Muldoon, O. T. , Nightingale, A. , Lowe, R. , Griffin, S. M. , McMahon, G. , Bradshaw, D. , & Borinca, I. (2023). Sexual violence and traumatic identity change: Evidence of collective post‐traumatic growth. European Journal of Social Psychology, 53(7), 1372–1382. 10.1002/ejsp.2979

[bjso12856-bib-0047] Munro, A. , Kovats, R. S. , Rubin, G. J. , Waite, T. D. , Bone, A. , Armstrong, B. , Beck, C. R. , Amlôt, R. , Leonardi, G. , & Oliver, I. (2017). Effect of evacuation and displacement on the association between flooding and mental health outcomes: A cross‐sectional analysis of UK survey data. The Lancet Planetary Health, 1(4), e134–e141. 10.1016/S2542-5196(17)30047-5 28944321 PMC5597543

[bjso12856-bib-0048] Nomura, S. , Parsons, A. J. Q. , Hirabayashi, M. , Kinoshita, R. , Liao, Y. , & Hodgson, S. (2016). Social determinants of mid‐ to long‐term disaster impacts on health: A systematic review. International Journal of Disaster Risk Reduction, 16, 53–67. 10.1016/j.ijdrr.2016.01.013

[bjso12856-bib-0049] Norris, F. H. , Friedman, M. J. , Watson, P. J. , Byrne, C. M. , Diaz, E. , & Kaniasty, K. (2002). 60,000 disaster victims speak: Part I. An empirical review of the empirical literature, 1981—2001. Psychiatry, 65(3), 207–239. 10.1521/psyc.65.3.207.20173 12405079

[bjso12856-bib-0050] Norris, F. H. , & Uhl, G. A. (1993). Chronic stress as a mediator of acute stress: The case of hurricane Hugo. Journal of Applied Social Psychology, 23(16), 1263–1284. 10.1111/j.1559-1816.1993.tb01032.x

[bjso12856-bib-0051] Ntontis, E. , Blackburn, A. M. , Han, H. , Stöckli, S. , Milfont, T. L. , Tuominen, J. , Griffin, S. M. , Ikizer, G. , Jeftic, A. , Chrona, S. , Nasheedha, A. , Liutsko, L. , & Vestergren, S. (2023). The effects of secondary stressors, social identity, and social support on perceived stress and resilience: Findings from the COVID‐19 pandemic. Journal of Environmental Psychology, 88, 102007. 10.1016/j.jenvp.2023.102007 37041753 PMC10079323

[bjso12856-bib-0052] Ntontis, E. , Drury, J. , Amlôt, R. , Rubin, G. J. , & Williams, R. (2019). What lies beyond social capital? The role of social psychology in building community resilience to climate change. Traumatology, 26(3), 253–265. 10.1037/trm0000221

[bjso12856-bib-0053] Ntontis, E. , Drury, J. , Amlôt, R. , Rubin, G. J. , & Williams, R. (2020). Endurance or decline of emergent groups following a flood disaster: Implications for community resilience. International Journal of Disaster Risk Reduction, 45, 101493. 10.1016/j.ijdrr.2020.101493

[bjso12856-bib-0054] Ntontis, E. , Drury, J. , Amlot, R. , Rubin, G. J. , Williams, R. , & Saavedra, P. (2020). Collective resilience in the disaster recovery period: Emergent social identity and observed social support are associated with collective efficacy, well‐being, and the provision of social support. British Journal of Social Psychology, 1–21, 1075–1095. 10.1111/bjso.12434 33340132

[bjso12856-bib-0055] Ntontis, E. , Drury, J. , Amlôt, R. , Rubin, G. J. , Williams, R. , & Saavedra, P. (2021). Collective resilience in the disaster recovery period: Emergent social identity and observed social support are associated with collective efficacy, well‐being, and the provision of social support. British Journal of Social Psychology, 60(3), 1075–1095. 10.1111/bjso.12434 33340132

[bjso12856-bib-0056] Ntontis, E. , Drury, J. , Amlôt, R. , Rubin, J. G. , & Williams, R. (2018). Emergent social identities in a flood: Implications for community psychosocial resilience. Journal of Community and Applied Social Psychology, 28(1), 3–14. 10.1002/casp.2329

[bjso12856-bib-0057] Ntontis, E. , Williams, R. , Luzynska, K. , Wright, A. , & Rousaki, A. (2024). Stressors and lessons for future support for healthcare staff facing adverse challenges: A systematic review of qualitative research conducted in the UK during the COVID‐19 pandemic. MedRxiv (Preprint). 10.1101/2024.04.16.24305910

[bjso12856-bib-0058] Ntontis, E. , & Zhang, L. (2024). Collective psychosocial resilience as a group process following flooding–How it arises and how groups can sustain it. In R. Williams , V. Kemp , K. Porter , T. Healing , & J. Drury (Eds.), Pandemics, major incidents and mental health. Cambridge University Press.

[bjso12856-bib-0059] Pearlin, L. I. (1989). The sociological study of stress. Journal of Health and Social Behavior, 30(3), 241–256. 10.2307/2136956 2674272

[bjso12856-bib-0060] Pearlin, L. I. , Aneshensel, C. S. , & Leblanc, A. J. (1997). The forms and mechanisms of stress proliferation: The case of AIDS caregivers. Journal of Health and Social Behavior, 38(3), 223–236. 10.2307/2955368 9343962

[bjso12856-bib-0061] Pearlin, L. I. , Mullan, J. T. , Semple, S. J. , & Skaff, M. M. (1990). Caregiving and the stress process: An overview of concepts and their measures. Gerontologist, 30(5), 583–594. 10.1093/geront/30.5.583 2276631

[bjso12856-bib-0062] Praharso, N. F. , Tear, M. J. , & Cruwys, T. (2017). Stressful life transitions and wellbeing: A comparison of the stress buffering hypothesis and the social identity model of identity change. Psychiatry Research, 247, 265–275. 10.1016/j.psychres.2016.11.039 27936438

[bjso12856-bib-0063] Sani, F. , Madhok, V. , Norbury, M. , Dugard, P. , & Wakefield, J. R. (2015). Greater number of group identifications is associated with healthier behaviour: Evidence from a Scottish community sample. British Journal of Health Psychology, 20(3), 466–481. 10.1111/bjhp.12119 25270579

[bjso12856-bib-0064] Stancombe, J. , Stokes, S. , Wood, A. , & Williams, R. (2024). How emergencies, incidents, disasters, and disease outbreaks affect people and healthcare practitioners. In R. Williams , V. Kemp , K. Porter , T. Healing , & J. Drury (Eds.), Major incidents, pandemics and mental health (1st ed., pp. 15–22). Cambridge University Press. 10.1017/9781009019330.007

[bjso12856-bib-0065] Stancombe, J. , Williams, R. , Drury, J. , Collins, H. , Lagan, L. , Barrett, A. , French, P. , & Chitsabesan, P. (2022). People's experiences of distress and psychosocial care following a terrorist attack: Interviews with survivors of the Manchester arena bombing in 2017. BJPsych Open, 8(2), e41. 10.1192/bjo.2022.2 35109959 PMC8867861

[bjso12856-bib-0066] Stancombe, J. , Williams, R. , Drury, J. , Hussey, L. , Gittins, M. , Barrett, A. , French, P. , & Chitsabesan, P. (2023). Trajectories of distress and recovery, secondary stressors and social cure processes in people who used the resilience hub after the Manchester arena bombing. BJPsych Open, 9(5), e143. 10.1192/bjo.2023.527 37550867 PMC10594089

[bjso12856-bib-0067] Stancombe, J. , Williams, R. , & Kemp, V. (2024). Facilitating psychosocial care for the public after major incidents and during pandemics. In R. Williams , V. Kemp , K. Porter , T. Healing , & J. Drury (Eds.), Major incidents, pandemics and mental health (1st ed., pp. 199–212). Cambridge University Press. 10.1017/9781009019330.030

[bjso12856-bib-0068] Stokols, D. (1985). A congruence analysis of human stress. Issues in Mental Health Nursing, 7(1–4), 35–64. 10.3109/01612848509009449 3854017

[bjso12856-bib-0069] Tempest, E. L. , English National Study on Flooding and Health Study Group , Carter, B. , Beck, C. R. , & Rubin, G. J. (2017). Secondary stressors are associated with probable psychological morbidity after flooding: A cross‐sectional analysis. European Journal of Public Health, 27(6), 1042–1047. 10.1093/eurpub/ckx182 29087460 PMC5881756

[bjso12856-bib-0070] Vignoles, V. L. , Jaser, Z. , Taylor, F. , & Ntontis, E. (2021). Harnessing shared identities to mobilize resilient responses to the COVID‐19 pandemic. Political Psychology, 42(5), 817–826. 10.1111/pops.12726 33821062 PMC8013210

[bjso12856-bib-0071] Wakefield, J. R. , Bowe, M. , Kellezi, B. , McNamara, N. , & Stevenson, C. (2019). When groups help and when groups harm: Origins, developments, and future directions of the “social cure” perspective of group dynamics. Social and Personality Psychology Compass, 13(3), e12440. 10.1111/spc3.12440

[bjso12856-bib-0072] Wang, Y. , Huang, Z. , & Kong, F. (2020). Parenting stress and life satisfaction in mothers of children with cerebral palsy: The mediating effect of social support. Journal of Health Psychology, 25(3), 416–425. 10.1177/1359105317739100 29129110

[bjso12856-bib-0073] Wang, Y. A. , & Rhemtulla, M. (2021). Power analysis for parameter estimation in structural equation modeling: A discussion and tutorial. Advances in Methods and Practices in Psychological Science, 4(1), 2515245920918253. 10.1177/2515245920918253

[bjso12856-bib-0074] Wilkinson, R. , & Pickett, K. (2010). The spirit level: Why equality is better for everyone. Penguin UK.

[bjso12856-bib-0075] Williams, R. , Kemp, V. J. , & Alexander, D. A. (2014). The psychosocial and mental health of people who are affected by conflict, catastrophes, terrorism, adversity and displacement. In J. M. Ryan , A. P. C. C. Hopperus Buma , C. W. Beadling , A. Mozumder , D. M. Nott , N. M. Rich , W. Henny , & D. MacGarty (Eds.), Conflict and catastrophe medicine (pp. 805–849). Springer. 10.1007/978-1-4471-2927-1_49

[bjso12856-bib-0076] Williams, R. , Ntontis, E. , Alfadhli, K. , Drury, J. , & Amlôt, R. (2021). A social model of secondary stressors in relation to disasters, major incidents and conflict: Implications for practice. International Journal of Disaster Risk Reduction, 63, 102436. 10.1016/j.ijdrr.2021.102436

[bjso12856-bib-0077] Williams, R. , Ntontis, E. , Drury, J. , Alfadhli, K. , & Amlôt, R. (2024). Primary and secondary stressors: The ways in which emergencies, incidents, disasters, disease outbreaks, and conflicts are stressful. In R. Williams , V. Kemp , K. Porter , T. Healing , & J. Drury (Eds.), Major incidents, pandemics and mental health (1st ed., pp. 42–48). Cambridge University Press. 10.1017/9781009019330.011

